# ^18^F-FDG PET/CT SUVmax in pleuropulmonary solitary fibrous tumors: can it be incorporated into risk classification systems for predicting recurrence?

**DOI:** 10.1186/s12890-025-03709-7

**Published:** 2025-07-04

**Authors:** İlteriş Türk, Mehmet Çetin, Necati Solak, Ali Can Kayaaslan, Nesrin Gürçay, Ebru Tatcı, Göktürk Fındık, Pınar Bıçakçıoğlu, Selim Şakir Erkmen Gülhan

**Affiliations:** 1 Department of Thoracic Surgery, University of Health Sciences, Atatürk Sanatoryum Training and Research Hospital, Ankara, Türkiye; 2 Department of Thoracic Surgery, Ankara Etlik City Hospital, Ankara, Türkiye; 3 Department of Pathology, University of Health Sciences, Atatürk Sanatoryum Training and Research Hospital, Ankara, Türkiye; 4 Department of Nuclear Medicine, Ankara Etlik City Hospital, Ankara, Türkiye; 5 Pınarbaşı Mah. Sanatoryum Cad. Atatürk Sanatoryum EAH B Blok2., Kat Göğüs Cerrahisi Kliniği, Ankara, Keçiören Türkiye

**Keywords:** Recurrence, Solitary fibrous tumor, Surgery, SUVmax

## Abstract

**Background:**

Several studies have been conducted to identify the parameters associated with the aggressive course of pleuropulmonary solitary fibrous tumors (SFT) following surgical treatment, and various classification systems have been proposed for assessing risk.

**Methods:**

The surgical outcomes of patients with pleuropulmonary SFT who underwent surgery between 2009 and 2024 at our center were retrospectively evaluated. Parameters of patients who experienced recurrence during follow-up were analyzed, and the risk classification systems proposed by Demicco were tested on our patient cohort. The pozitron emision tomography/ computed tomography (PET/CT) standart uptake value(SUVmax), which was significantly associated with recurrence, was incorporated into the modified Demicco classification, and a new classification model was developed and compared with existing models.

**Results:**

Among the 79 included patients, 21.5% had intraparenchymal tumors, which were excised along with various parenchymal resections, while pleural tumors underwent mass excision. The postoperative follow-up period was 108.16 ± 44.09 months, during which 7.6% of patients experienced recurrence. Tumor size (*p* = 0.023), mitotic index (*p* < 0.001), presence of necrosis (*p* = 0.007), and PET/CT SUVmax value (*p* = 0.005) were found to be significantly associated with recurrence. The PET/CT SUVmax value, which was not included in Demicco’s classifications, ranged from 1.12 to 7.98, with a cutoff value of 4.50. The addition of SUVmax to the modified Demicco classification categorized all patients with recurrence into the high-risk group, and the new classification model strongly differentiated recurrence (*p* < 0.01, 100% sensitivity, 71.43% specificity).

**Conclusion:**

The incorporation of SUVmax into the modified Demicco classification system for pleuropulmonary SFT provides a more significant prediction of recurrence.

## Introduction

Solitary fibrous tumor (SFT) is a rare primary pleural tumor originating from mesenchymal cells. Although these tumors are typically benign, the definitive treatment is considered to be surgical excision. Recurrence and metastasis can be observed in certain proportions during postoperative follow-up [1].

Several studies have been conducted to predict the potential for aggressive behavior in SFT, and various classification systems have been proposed. In 2002, Perrot and colleagues divided pleural SFT into five stages based entirely on histopathological data [2]. In 2012, Demicco and colleagues, based on the classification previously proposed by England [3], developed risk prediction models that included extrathoracic SFT and aimed to predict metastatic potential and poor survival associated with the disease [4]. In 2017, this model was modified with the addition of the"necrosis"parameter, and a modified classification system was proposed. In this system, pathological parameters remained central as in Perrot’s system, but demographic parameters such as"age"were also included in the scoring [5]. In addition to these models, Tapias’ study, which incorporated more histopathological parameters, tumor location, and bleeding status, can also be mentioned [6]. A recent study found that the modified Demicco classification not only better identified aggressive SFT patients but also had easier clinical applicability [7].

Our study aims to test the parameters associated with recurrence and the applicability of the risk classification systems proposed by Demicco using data from patients with pleuropulmonary SFT who underwent surgery at our center.

## Materials and methods

After obtaining local ethics committee approval, this retrospective study included patients diagnosed with pleuropulmonary SFT who underwent surgery at our clinic between 2009 and 2024. Demographic, radiological, pathological, and prognostic data of the patients were evaluated. Only SFT cases with negative surgical margins were included in the study. Patients whose data could not be fully accessed or those with positive surgical margins in postoperative pathology reports were excluded from the study. The study was conducted in accordance with the Declaration of Helsinki on Human Rights.

In cases of intraparenchymal SFT, anatomical and non-anatomical lung resections were performed. For SFT originating from the visceral or parietal pleura, mass excision was carried out. Mass excision in visceral pleura-originating SFT involved removing the tumor along with minimal lung tissue, while for parietal pleura-originating SFT, excision was done according to the borders of the pedicle of the tumor.

The parameters to be evaluated in relation to recurrence were determined from the data of the patients who underwent surgery. After these parameters were identified, the Demicco and modified Demicco classification systems were applied to our patients, and the patients were categorized into low, moderate, and high-risk groups. The parameters and scoring of the classification systems are mentioned in Table 1 [4, 5].

**Table 1:** Demicco and modified demicco classification criteria

Classification	Criterion	Score	Criterion	Score	Criterion	Score	Criterion	Score	Risk	Total Score
Demicco (2012)	Age < 55 ≥ 55	0 1	Size < 5 cm 5–10 cm 10–15 cm ≥ 15 cm	0 1 2 3	Mitosis 0 1–3 ≥ 4	0 1 2			Low Mod High	0–2 3–4 5–6
Modified Demicco (2017)	Age < 55 ≥ 55	0 1	Size < 5 cm 5–10 cm 10–15 cm ≥ 15 cm	0 1 2 3	Mitosis 0 1–3 ≥ 4	0 1 2	Necrosis None Present	0 1	Low Mod High	0–3 4–5 6–7

In addition to the parameters proposed by the classification systems, it was determined that the SUVmax value from PET/CT was a significant parameter for recurrence. The SUVmax value, not included in the existing classification systems, was scored based on its cutoff value. This value was incorporated into the modified Demicco classification as a score of zero for values below the cutoff and a score of one for values above it, creating a new model. This new model was then compared to the Demicco and modified Demicco classifications.

PET/CT imaging (Biograph LSO HI-REZ PET/CT; Siemens, Medical Solutions, Knoxville, TN) was performed 45–60 min after the intravenous administration of 18 F-FDG at a dose of 0.15 mCi/kg. Prior to injection, blood glucose levels were confirmed to be < 200 mg/dL. All patients fasted for at least six hours before the procedure. Computed tomography (CT) images were acquired from the vertex of the skull to the proximal femur, followed by PET imaging in 6–8 bed positions (3 min per position). CT data were used for attenuation correction. PET images were reconstructed using the ordered-subsets expectation maximization (OSEM) algorithm with four iterations and eight subsets. A Gaussian post-filter was applied, resulting in a transaxial spatial resolution of 5 mm at full width at half maximum. The voxel size of the reconstructed PET images was 3.6 × 3.6x4 mm^3^. For each lesion, the maximum standardized uptake value (SUVmax) and longest diameter were recorded. SUVmax values were automatically calculated by delineating the region of interest around each lesion.

### Statistical analysis

Data were analyzed using IBM SPSS Statistics Standard Concurrent User V 27 (IBM Corp., Armonk, New York, USA). Descriptive statistics were presented as number (n), percentage (%), mean ± standard deviation (mean ± SD) for age, and median ± standard error (median ± SE) for follow-up and recurrence development times. Postoperative follow-up patients were divided into two groups: those with recurrence and those without recurrence. Since the number of patients with recurrence was small, continuous numerical variables were compared using the Mann–Whitney U test between the two groups. Distribution comparisons of categorical variables between groups were assessed using Pearson’s Chi-squared test and Fisher's exact test. Recurrence development was calculated using the Kaplan Meier method and compared between groups using the Log rank test. Cox regression analysis was used for Hazard Ratio (HR) values. Logistic regression analysis was used to obtain predictive values (prediction) from categorical variables for the Demicco, modified Demicco, and new classification systems. The cutoff value for cases and comparisons of diagnostic performance for two or more parameters were evaluated using the ROC curve analysis method. A p-value of < 0.05 was considered statistically significant.

## Results

**Fig. 1:**
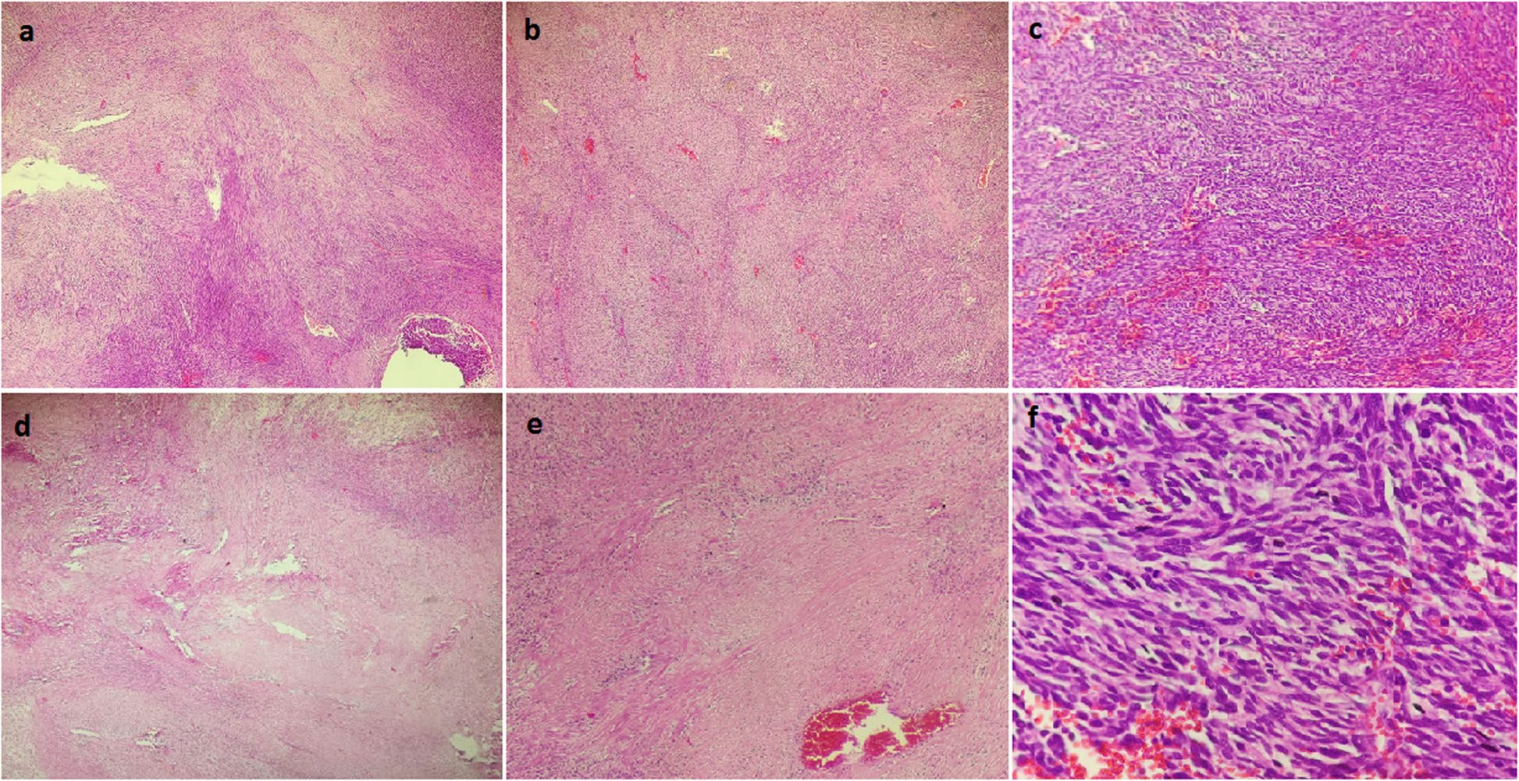
**a** SFT consisting of spindle cells with a uniform appearance, containing hypocellular and hypercellular areas in a storiform pattern (H&E, X100) **b** Spindle cells with uniform appearance (H&E, X100) **c** area of hypercellular appearance (H&E, X200) **d** Necrosis (H&E, X100) **e** Necrosis (H&E, X200) **f** Mitosis (H&E, X400)

A total of 79 pleuropulmonary SFT patients were included in the study, of which 46 (58.2%) were male. Seventeen patients (21.5%) had intraparenchymal SFT, and anatomical/non-anatomical lung resection was performed in these cases. Among the intraparenchymal cases, 12 were located in the lower lobe, 3 in the upperlobe, and 2 in the middle lobe. Data on patient age, gender, tumor size, PET/CT SUVmax value, tumor location, operation, pathology, and hospitalization time are shown in Table 2. Figure 1 shows the histopathological features of a SFT in a patient.

**Table 2:** Patient variables

Parameter	Values		
	Number (n/%)	Mean ± Standard Deviation	Median (min -max)
Age	79 (%100)	57,94 ± 11,49	59 (34–82)
Gender			
Male	46 (%58,2)		
Female	33 (%41,8)		
Tumor Size (mm)	79 (%100)		90 (15- 300)
Tumor PET/CT SUVmax	69 (%87,3)	3,64 ± 1,30	
Tumor Location			
Right	41 (%51,9)		
Left	38 (%48,1)		
Surgical Approach			
Thoracotomy	55 (%69,6)		
VATS	24 (%30,4)		
Type of Surgery			
Tumor Excision	62 (%78,5)		
Wedge Resection	8 (%10,1)		
Segmentectomy	1 (%1,3)		
Lobectomy	8 (%10,1)		
Additional Procedures			
None	74 (%93,7)		
Decortication	2 (%2,5)		
Diaphragm Resection	2 (%2,5)		
Rib Resection	1 (%1,3)		
Mitosis Count (per 10X magnificationfield)	79 (%100)		3 (0–30)
Necrosis			
None	43 (%54,4)		
Present	36 (%45,6)		
Lymph Node Dissection			
Not Performed	63 (%79,7)		
Performed/Negative	16 (%20,3)		
Length of Hospital Stay (days)	79 (%100)		5(1–15)

**Fig. 2:**
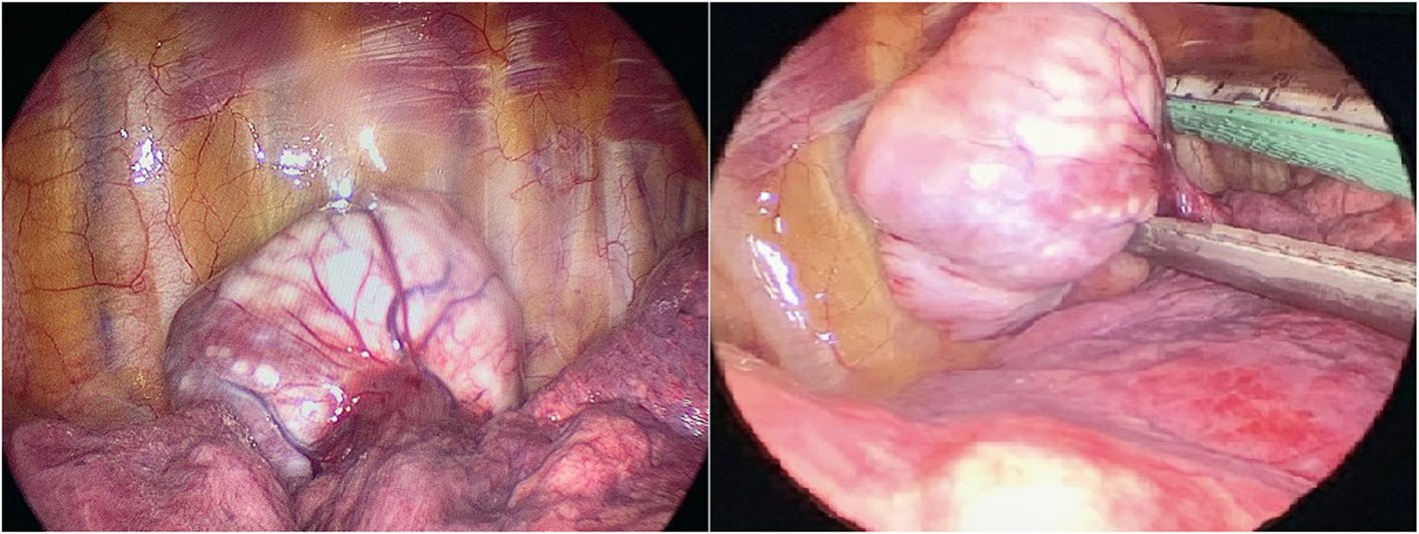
VATS can be easily applied in the treatment of small-sized SFTs. In a 44-year-old female patient, a 50-mm pleural SFT was excised using a stapler along with a small portion of lung tissue. The patient was discharged on the 3rd postoperative day

No surgical mortality was observed in the patient cohort. The postoperative follow-up duration was 108.16 ± 44.09 months. Mortality occurred in only one patient during the 9 th year of follow-up. Twenty-four cases (30.4%) were operated on via video-assisted thoracoscopicsurgery (VATS). In Fig. 2, intraoperative images of a patient operated via VATS are shown. The average tumor size in the VATS group was 40 mm (min = 15, max = 74), while in the thoracotomy group, it was 120 mm (min = 17, max = 300), showing a statistically significant difference (*p* < 0.001). Median hospital stay was statistically significantly shorter in the VATS group than in the thoracotomy group (4 days and 6 days, respectively, *p* < 0.01). Nine patients who underwent thoracotomy experienced various operative complications (11.4%). The most common complications were intraoperative massive bleeding and postoperative secondary pleural effusion requiring drainage, each occurring in three patients (3.8%). Additionally, one patient had a wound infection, one had pneumonia, one had pulmonary thromboembolism, and one had prolonged air leakage. No complications were observed in the VATS group. PET/CT data were available for 69/79 patients. The parameters of the patients and their relationship to recurrence are shown in Table 3, The relationship between SUVmax and other clinicopathological features is shown in Table 4.

**Table 3:** Comparison of patient variables according to recurrence status

Parameter	Recurrence		
	Present	None	p
Gender			
Male	5 (%10,9)	41 (%89,1)	0,196
Female	1 (%3,3)	32 (%96,7)	
Age	63 (min:36 – max:82)	54 (min:34 – max:81)	0,266
Side			
Right	4 (%9,8)	37 (%90,2)	0,375
Left	2 (%5,9)	32 (%84,1)	
Intraparenchymal			
Yes	1 (%5,9)	16 (%94,1)	0,617
No	5 (%8,7)	57 (%91,3)	
Tumor Size (mm)	135 (min:40 – max: 300)	55 (min:15 – max: 200)	**0,023**
Tumor PET/CT SUVmax	4,08 (min: 2,13 – max: 7,98)	2,99 (min: 1,12 – max: 6,22)	**0,005**
Necrosis			
Yes	6 (%16,7)	30 (%83,3)	**0,007**
No	0 (%0)	43 (%100)	
Mitosis Count (10X magnification)	5 (min: 0 – max: 30)	1 (min: 0 – max: 8)	** < 0,001**

**Table 4:** Relationship between SUVmax and clinicopathologic factors

Parameter	**SUVmax**		
	Number (n)	Median (min–max)	p
**Age**			
≤ 55	29	3,33 (1,66–6,97)	0,956
> 55	40	3,68 (1,12–7,98)	
**Necrosis**			
Var	33	4,08 (2,13–7,98)	** < 0,001**
Yok	36	2,99 (1,12–6,22)	
**Mitosis count**			
0	11	2,98 (1,64–4,58)	
1–3	27	3,04 (1,12–7,98)	** < 0,001**
≥ 4	31	3,78 (1,63–6,97)	
**Tumor size (cm)**			
0–4,9	14	2,51 (1,12–3,84)	
5- 9,9	23	3,13 (2,01–4,92)	** < 0,001**
10–14,9	16	4,14 (1,70–7,98)	
≥ 15	16	4,00 (2,13–6,97)	

**Fig. 3:**
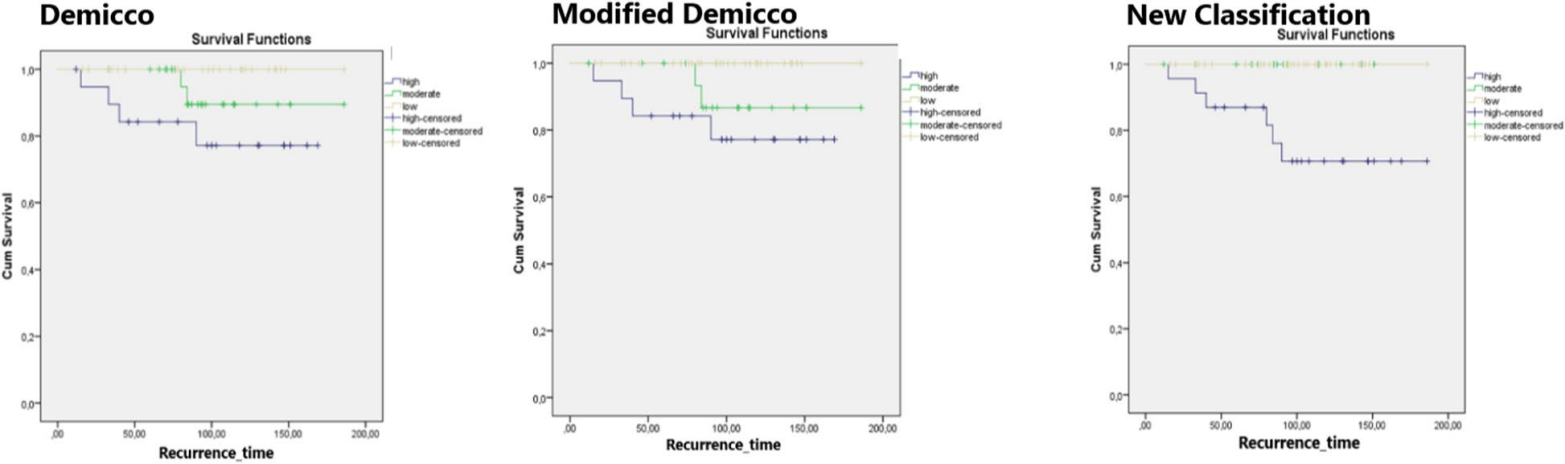
Evaluation of recurrence according to classifications with Kaplan–Meier analysis. Unlike Demicco (*p* = 0,068) and Modified Demicco (*p* = 0,05) classifications, all recurrences are seen in the high risk group in the New Classification and the difference is statistically significant (*p* = 0,003). According to Cox regression analysis, Hazard Ratio (HR) for Demicco classification was 0.320 (95% Confidence Interval (CI): 0.790–1.289), HR for modified Demicco classification was 0.325 (95% CI: 0.840–1.261), and HR for the new classification was 0.090 (95% CI: 0.002–5.375)

The cut-off value for the SUVmax was calculated as 4.50. This value was able to distinguish recurrence with 83.3% sensitivity and 82.5% specificity. For the SUVmax value, not included in the Demicco and modified Demicco systems, a score of 0 was given for values below 4.50 and a score of 1 for values above 4.50. Patients were categorized into low risk (0–3 points), moderate risk (4–5 points), and high risk (6–8 points) groups. The number of patients in each risk group according to the classification systems is shown in Table 5. Kaplan–Meier analyses performed on the classification groups for recurrence are shown in Fig. 3.

**Table 5:** Distribution of risk groups according to Demicco, Modified Demicco, and the New Classification

	Low risk	Moderate risk	High risk
Demicco	23/69	26/69 (2 recurrences)	20/69 (4 recurrences)
Modified Demicco	31/69	19/69 (2 recurrences)	19/69 (4 recurrences)
New Classification	30/69	15/69	24/69 (6 recurrences)

**Fig. 4:**
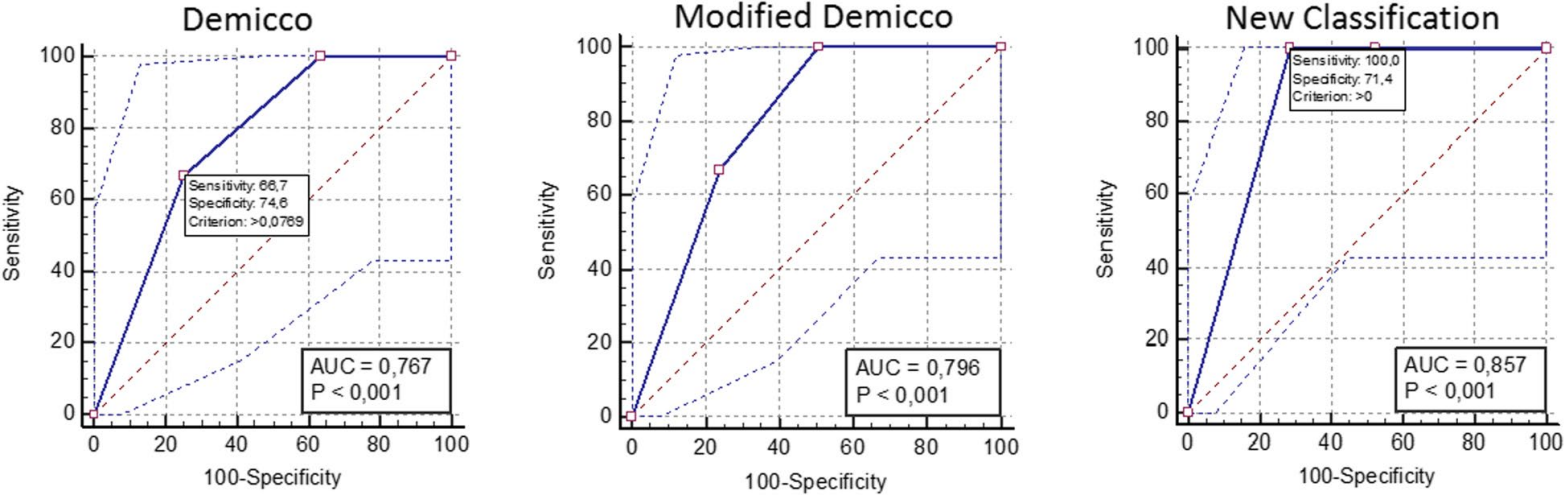
All three classification systems were found to provide statistically significant results in distinguishing recurrence

**Fig. 5:**
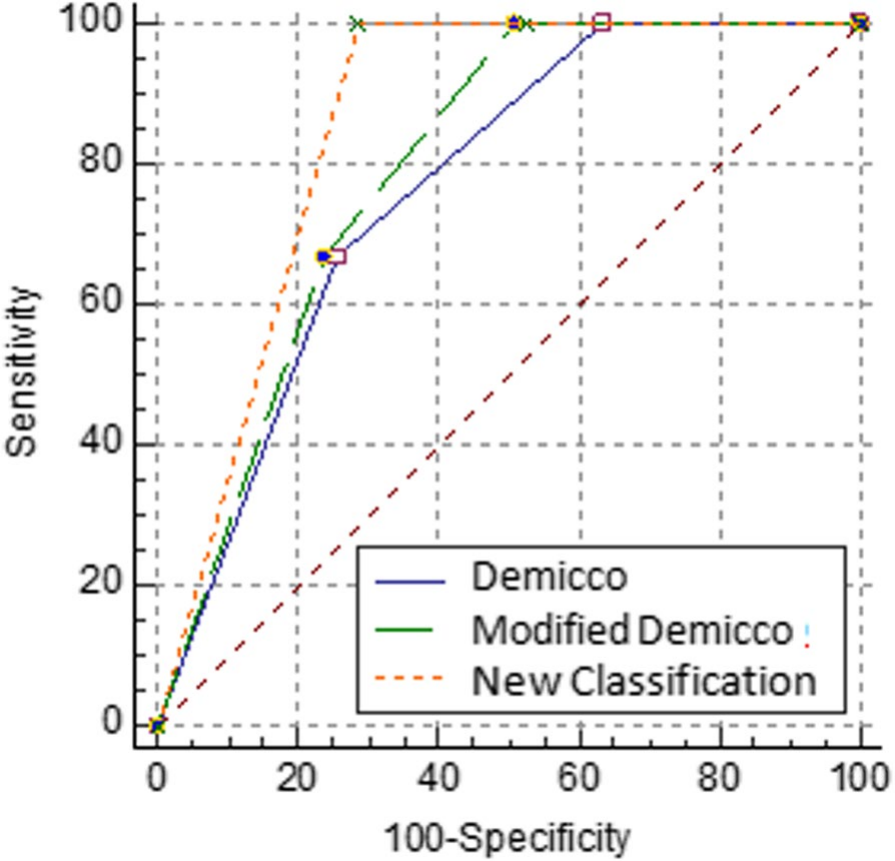
While the difference in the area under the ROC curves between the Demicco and modified Demicco systems was not statistically significant, the new classification system distinguishes recurrence more effectively than both

Logistic regression analysis was performed to obtain predictive values for the Demicco, modified Demicco, and new classification systems based on categorical variables. All three classification systems were statistically significant in distinguishing recurrence (all *p* < 0.001) (Fig. 4). When the systems were compared, it was observed that the Demicco and modified Demicco systems showed similar performance (*p* = 0.152). The new classification system performed beter than both Demicco (ROC curve area difference statistically significant, AUC Difference = 0.0899, *p* = 0.033) and modified Demicco (AUC Difference = 0.0608, *p* = 0.045) (Fig. 5). The new classification system demonstrated strong performance in distinguishing recurrence with 100% sensitivity and 71.43% specificity (Table 6).

**Table 6:** ROC Analysis of Classification Systems for Recurrence

Variable	*AUC*	*se*	*p*	Asymptotic 95% Confidence Interval		Sensitivity	Specificity
				L. Bound	U. Bound		
Demicco	0,767	0,074	< 0,001	0,650	0,861	66,67	74,60
Modified Demicco	0,796	0,063	< 0,001	0,682	0,884	100,0	49,21
New Classification	0,857	0,028	< 0,001	0,752	0,930	**100,0**	**71,43**

*AUC*: Area under the curve, *CI*: Confidence interval, *se*: Standard error

## Discussion

The SUVmax value of SFT on PET/CT is significantly correlated with recurrence. When the SUVmax data is integrated into the modified Demicco classification system, which has been tested for clinical applicability and accuracy, recurrence can be predicted with high success. Similarly, Herder et al. (2005) demonstrated that adding the PET/CT tumor SUVmax value to the Mayo Clinic model, a system used for predicting the malignancy of pulmonary nodules, could provide more accurate predictions than the previous model [8]. In the validation study by El-Ameri et al., although other nodule risk prediction models were able to distinguish benign from malignant nodules, the model proposed by Herder showed beter accuracy [9]. A recent study on the Turkish populational so found that the Herder model, which includes the SUVmax value, performed better in distinguishing malignancy than the Brock and Mayo models, which do not include this parameter [10]. For SFT, the SUVmax value on PET/CT has also been shown in previous literature to be a good indicator for predicting an aggressive course. In a study by Bove et al., involving 107 patients from two centers in Italy, SUVmax successfully predicted recurrence along with a high mitotic index [11]. Another study conducted in Turkey, as well as research in China, demonstrated that SUVmax could significantly predict the malignancy status of previously pathologically benign and malignant groups with a smaller sample size [12, 13]. Based on these results from pulmonary nodules, our study suggests that evaluating the SUVmax value in conjunction with the parameters from the risk classification models for SFT can improve the prediction of recurrence. Larger and multicenter studies may re-test the cut-off value for SUVmax in SFT.

Chemotherapy has limited applicability in pleuropulmonary SFT, and radiotherapy is usually considered after R1 or R2 resection. It is well established that the definitive treatment is surgical [14]. The main focus of studies in this field is not on the type of treatment but on patients with recurrence and metastasis after surgical treatment. In a large study including 147 pleuropulmonary SFT patients, Reisenauer reported a recurrence and metastasis rate of 10.2% [7]. In a meta-analysis involving 23 studies and 1262 patients, the recurrence rate after surgery for pleural SFT was calculated to be 9% [15]. Although the studies included in this analysis had significantly varying follow-up durations, we observed a similar recurrence rate of 7.6% over a follow-up period of 108.16 ± 44.09 months in our patient group.

Video-assisted thoracoscopic surgery (VATS) can be safely and effectively used in appropriate pleuropulmonary SFT cases. The main limitation of VATS in SFT is not the surgical technique itself, but the large size that some tumors may reach. In a study by Liu et al., in which 21 SFT cases were operated on via VATS, it was reported that in 71.4% of the cases, surgery was completed via VATS, while in 6 cases, the procedure was converted to minithoracotomy or posterolateral thoracotomy [16]. A study based on the experience of the Chinese National Cancer Center included 82 patients, 22 of whom underwent VATS. The tumor sizes in the VATS group were significantly smaller than those in the thoracotomy group. Furthermore, VATS was associated with a shorter operation time, less intraoperative blood loss, and a shorter hospital stay [17]. In our study, 24 patients (30.4%) were operated on via VATS. We observed that tumor size and hospital stay were significantly lower in the VATS group compared to the thoracotomy group, and no surgical complications or recurrences were observed in the VATS group. We hypothesize that the lack of recurrence in the VATS group is related to the smaller tumor sizes. Were commend the preferential use of VATS for smaller tumors.

Pleuropulmonary SFT, although encountered at varying rates in different studies, may show intraparenchymal characteristics. In a study by Zhang et al., 17 out of 50 patients (34%) had pulmonary SFT [18]. Another study conducted in Türkiye reported this rate as 20% [19]. A series of 52 cases analyzing pulmonary SFT highlighted that the disease most commonly presents in the left lower lobe and tumors are typically 1–10 cm in size [20]. In our cohort, 21.5% (17/79) of the pleuropulmonary SFT were located intraparenchymally. Among these, 82.4% (14/17) were between 1 and 10 cm in size, and 47.1% of these tumors were located in the left lower lobe. Our findings are consistent with the literature.

Our study has limitations due to the rarity of SFT cases, leading to a relatively small sample size, the lack of survival and metastasis analysis due to the very low mortality in our cohort, and the retrospective nature of the study. In addition, the limited availability of PET/CT in all centers and the variability related to the technical specifications of PET/CT devices may affect the implementation of the new classification.

In conclusion, our study shows that adding the SUVmax value to the accepted modified Demicco classification can significantly improve the prediction of recurrence in pleuropulmonary SFT despite the limitations. Larger, multicenter studies may both validate our findings and assess the predictive role of PET/CT for metastasis and survival.

## Data Availability

The datasets used and analysed during the current study are available from the corresponding author on reasonable request.
